# Induction of Boosted Immune Response in Mice by Leptospiral Surface Proteins Expressed in Fusion with DnaK

**DOI:** 10.1155/2014/564285

**Published:** 2014-07-06

**Authors:** Marina V. Atzingen, Dunia Rodriguez, Gabriela Hase Siqueira, Luciana C. C. Leite, Ana L. T. O. Nascimento

**Affiliations:** ^1^Centro de Biotecnologia, Instituto Butantan, Avenida Vital Brazil 1500, 05503-900 São Paulo, SP, Brazil; ^2^Programa Interunidades em Biotecnologia, Instituto de Ciências Biomédicas, USP, Avenida Professor Lineu Prestes 1730, 05508-900 São Paulo, SP, Brazil

## Abstract

Leptospirosis is an important global disease of human and veterinary concern. Caused by pathogenic *Leptospira*, the illness was recently classified as an emerging infectious disease. Currently available veterinarian vaccines do not induce long-term protection against infection and do not provide cross-protective immunity. Several studies have suggested the use of DnaK as an antigen in vaccine formulation, due to an exceptional degree of immunogenicity. We focused on four surface proteins: rLIC10368 (Lsa21), rLIC10494, rLIC12690 (Lp95), and rLIC12730, previously shown to be involved in host-pathogen interactions. Our goal was to evaluate the immunogenicity of the proteins genetically fused with DnaK in animal model. The chosen genes were amplified by PCR methodology and cloned into pAE, an *E. coli* vector. The recombinant proteins were expressed alone or in fusion with DnaK at the N-terminus. Our results demonstrate that leptospiral proteins fused with DnaK have elicited an enhanced immune response in mice when compared to the effect promoted by the individual proteins. The boosted immune effect was demonstrated by the production of total IgG, lymphocyte proliferation, and significant amounts of IL-10 in supernatant of splenocyte cell cultures. We believe that this approach could be employed in vaccines to enhance presentation of antigens of *Leptospira* to professional immune cells.

## 1. Introduction

Leptospirosis is a widespread zoonosis caused by pathogenic* Leptospira* spp. that is transmitted from reservoir hosts to humans through water and soil contaminated with their urine [1]. In the urban settings, due to the disorganized growing population allied to the poor sanitation conditions and the increasing presence of brown rats, the disease has become a major public health problem. Symptoms of the disease include fever, vomiting, headache, diarrhea, and abdominal and generalized muscle pain. Progression to multiorgan system complications, known as Weil's syndrome, occurs in 5–15% of cases, with mortality rates of 5–40% [2].

The best strategy to fight leptospirosis is through the implementation of prophylactic measures. However, available vaccines are the veterinarian ones, based on inactivated whole cell or membrane preparations of pathogenic leptospires. They confer protective responses frequently through the induction of antibodies against leptospiral lipopolysaccharide [1, 2]. These vaccines, however, do not induce long-term protection against infection and do not provide cross-protective immunity against leptospiral serovars not included in the vaccine preparation [3, 4]. Cuba, France, and China have licensed vaccines for human [5–7]. Due to the large number of leptospiral serovars, conserved and protective antigens are being pursued.

DnaK is a member of Hsp70 family that appears to play an important role in the innate and adaptive immune responses. It is involved in receptor-mediated antigen internalization by sentinel antigen-presenting cells (APCs), stimulation of production of various cytokines, and maturation of dendritic cells [8]. Several studies have suggested the use of DnaK as an antigen in the formulation of vaccines, due to its high degree of immunogenicity (humoral and cellular) and the ability to stimulate T cells to produce IL10 [9–15].

By data mining the genome sequences of* L. interrogans* we have selected and characterized several leptospiral proteins as novel adhesins [16] and cellular adhesion molecules (CAM) inducers [17]. Among them were the adhesins Lsa21 [18], the C-terminus region of Lp95 [19], and two proteins rLIC12730 and rLIC10494. The two preceding proteins had their immunogenic and immunoprotective activities evaluated in hamsters and have shown only modest performance [20].

Thus, we decided to take advantage of the HSP DnaK immunogenic properties and employ it to genetically combine with these four leptospiral genes LIC10368, LIC10494, LIC12730, and LIC12690. We aimed to evaluate if the fused DnaK-leptospiral proteins had their immunogenic activities boosted compared to the recombinant protein alone. We describe in this work, the in-frame cloning, protein expression, and characterization of the DnaK-leptospiral recombinant fusion proteins and their immunogenic evaluation in mice.

## 2. Material and Methods

### 2.1. Cloning, Expression, and Purification of Recombinant Proteins

Amplification of the CDSs was performed by PCR from total* L. interrogans* serovar Copenhageni strain Fiocruz L1-130 genomic DNA using complementary primer pairs listed in Table 1.

PCR fragments were cloned into pGEM-T easy vector (Promega) and transformed into* E. coli* DH5*α* subcloned into the pAE expression vector [21], which allow the expression of recombinant proteins with a minimal 6X His-tag at the N-terminus. DnaK gene (LIC10524) amplified with 2X (Gly-Pro) tag at C-terminus was cloned into pAE vector at BamHI and PvuII restriction sites, while the LIC10368, LIC10494, LIC12690, and LIC12730 genes were cloned into pAE vector at BamHI and NcoI restriction sites. For the DnaK genetically fused with LIC genes, in-frame cloning was obtained by digesting the pAE-LIC gene with PvuII and NcoI restriction sites and ligated into pAE-DnaK plasmid at the same restriction sites. The genetically fused DnaK-LIC genes will have a flexible hinge between DnaK and leptospiral genes [22]. All cloned sequences were confirmed by DNA sequencing with an ABI 3100 automatic sequencer (PE Applied Biosystems, Foster city, CA).

Protein expression was achieved in* E. coli* BL21 (SI) strain by the action of T7 DNA polymerase under control of the osmotically induced promoter* proU* [23].* E. coli* BL21 (SI) containing recombinant plasmids were grown at 30°C in Luria-Bertani (LB) broth without NaCl and with 100 *μ*g/mL ampicillin with continuous shaking until an optical density at 600 nm of 0.6 to 0.8 was reached. Recombinant protein synthesis was induced by the addition of 300 mM NaCl. After three hours, the cells were harvested by centrifugation, the bacterial pellets resuspended in lysis buffer (20 mM Tris-HCl (pH 8.0), 200 mM NaCl, 100 *μ*g/mL of lysozyme, 2 mM phenylmethylsulfonyl fluoride (PMSF), and 1% Triton X-100). The bacterial cell pellets were lysed on ice with the aid of ultrasonic cell disruptor (Sonifier 450, Branson, USA). The bacterial lysate was centrifuged at 3,000 ×g for 10 min at 4°C. The pellets were resuspended in buffer containing 8 M urea; 20 mM Tris-HCl (Ph 8.0), 500 mM NaCl, and 5 mM imidazol. The recombinant proteins were recovered from the insoluble fraction except rDnaK and rLIC12730 that were recovered from the soluble fraction. All proteins were purified through Ni^2+^-charged beads of chelating fast-flow chromatographic column (GE Healthcare). The insoluble proteins were refolded on-column by gradually removing urea (8–0 M). The contaminants were washed away with low imidazole concentration and the recombinant proteins were eluted in 20 mM Tris-HCl (pH 8.0), 500 mM NaCl, and 500 mM imidazole. The efficiency of the purification was evaluated by 12% SDS-PAGE. The purified recombinant protein were extensively dialyzed against PBS (pH 7.4) glycine solution (wt/vol: 0.1%), at the proportion of 10 mL of protein per 1,000 mL of buffer, with at least five changes of buffer every 4 h for 48 h.

### 2.2. Circular Dichroism Spectroscopy

Recombinant proteins were dialyzed against sodium phosphate buffer (pH 7.4), except rLsa21 and rLp95 that were dialyzed against 100 mM Tris-HCl (pH 12.0), 500 mM NaCl. Circular dichroism (CD) spectroscopy measurements were performed at 20°C using a Jasco J-810 spectropolarimeter (Japan Spectroscopic, Tokyo, Japan) equipped with a Peltier unit for temperature control. Far-UV CD spectra were measured using a 1 mm-path-length cell at 0.5 nm intervals. The spectra were presented as an average of five scans recorded from 185 to 260 nm. The residual molar ellipticity is expressed in degree × cm^2^ per decimole. The data were submitted to CAPITO CD analysis program http://capito.nmr.fli-leibniz.de/index.php [24].

### 2.3. Mice Immunization

Five female BALB/c mice (4–6 weeks old) were immunized subcutaneously with 10 *μ*g of the recombinant proteins adsorbed in 10% (vol/vol) of Alhydrogel (2% Al(OH)_3_, Brenntag Biosector, Denmark), used as adjuvant. Two subsequent booster injections were given at 2-week intervals with the same recombinant proteins preparation. Negative-control mice were injected with PBS plus Alhydrogel. Two weeks after each immunization, the mice were bled from the retroorbital plexus to evaluate antibody response. The animals were then sacrificed, 45 days after the first inoculation, to isolate splenocytes for lymphocyte proliferation and cytokine profiles in response to prime boosted antigen.

### 2.4. ELISA for Detection of Mouse IgG Antibodies

For isotype determination, total IgG (immunoglobulin G), IgG1, and IgG2a were measured by incubation of pooled mice sera with recombinant protein followed by incubation with horseradish peroxidase- (HRP-) conjugated anti-mouse total IgG (1 : 5,000) or goat anti-mouse IgG1 or IgG2a (1 : 2,000) followed by incubation with HRP-conjugated anti-goat IgG (1 : 10,000). The optical density at 492 nm values exhibited by different dilution of mice sera were compared to a curve generated by coating the plates with different concentrations of mice total IgG, IgG1, or IgG2a.

### 2.5. Western Blotting for Identification of the Proteins Either Alone or in Fusion with DnaK

Aliquots (1 *μ*g) of each recombinant protein were subjected to SDS-PAGE and transferred to nitrocellulose membranes (Hybond ECL; GE Healthcare). Membranes were blocked with 10% nonfat dried milk in PBS containing 0.05% Tween 20 (PBS-T) and then incubated with anti-DnaK-Lsa21, anti-DnaK-rLIC10494, anti-DnaK-Lp95, or anti-DnaK-rLIC12730 mouse serum, all at 1 : 20,000 dilution in 5% nonfat dried milk/PBS-T for 2 h at room temperature. After washing, the membrane was incubated with HRP-conjugated anti-mouse IgG (1 : 5,000; Sigma) in 5% nonfat dried milk/PBS-T for 1 h. The bands were revealed with ECL reagent kit chemiluminescence substrate (GE Healthcare).

### 2.6. Lymphoproliferation Assay

At the end of the immunization protocols, BALB/c mice were sacrificed and their spleens were aseptically removed and suspended in RPMI culture medium (Roswell Park Memorial Institute Medium-RPMI-1640 medium). Spleens were macerated and after erythrocytes lysis splenocytes were resuspended in 1 mL of RPMI containing 10% fetal bovine serum and counted after staining with 0.4% trypan blue for viability. Spleen cells (5 × 10^5^ cells/well) were plated in triplicate in a 96-well flat bottom cell culture plates (Costar, Corning). Spleen cells were stimulated with 5 *μ*g/mL of Concanavalin A—Con A (Sigma), employed as positive control, 5 *μ*g/mL of recombinant protein, or culture medium alone, used as negative control. Cells were cultured for 48 h at 37°C and 5% CO_2_ in a humidified atmosphere and proliferative rates were determined as a function of DNA synthesis, measured by the incorporation of bromodeoxyuridine (BrdU) by BrdU ELISA colorimetric kit (Roche Diagnostic, Indianapolis, IN) according to the manufacturer's instructions. Stimulation Index (S.I.) was calculated as the ratio between the mean OD of cells, from DnaK-leptospiral proteins immunized mice, stimulated with the same fusion proteins and the mean OD of cells from mice immunized with the protein, stimulated with the same leptospiral protein.

### 2.7. Cytokine Production Evaluation

For analysis of secreted cytokines, spleen cells from surface proteins and from DnaK fusion proteins immunized animals were isolated and cultured as described above, except that the culture was made in 24 well tissue culture plates, each well containing 5 × 10^6^ cells. After 48 h, cell-free culture supernatants were collected and stored for short-term at −20°C. IL-4, IL-10, IFN-gamma, and TNF-alpha were measured by ELISA (PreproTech) according to the manufacturer's instructions.

### 2.8. Ethics of Animal Experimentation

All animal studies were approved by the Ethic Committees of the Instituto Butantan, São Paulo, Brazil, under the protocol number 576/09. This Committee adopts the guidelines of the Brazilian College of Animal Experimentation.

### 2.9. Statistical Analysis

All results are expressed as means ± SD. Student's paired *t*-test was used to determine the significance of differences between means, and *P* < 0.05 was considered as statistically significant.

## 3. Results

### 3.1. Construction of Genetically* in-Frame* Fusion of Dnak with Surface Protein Genes

**Figure 1:**
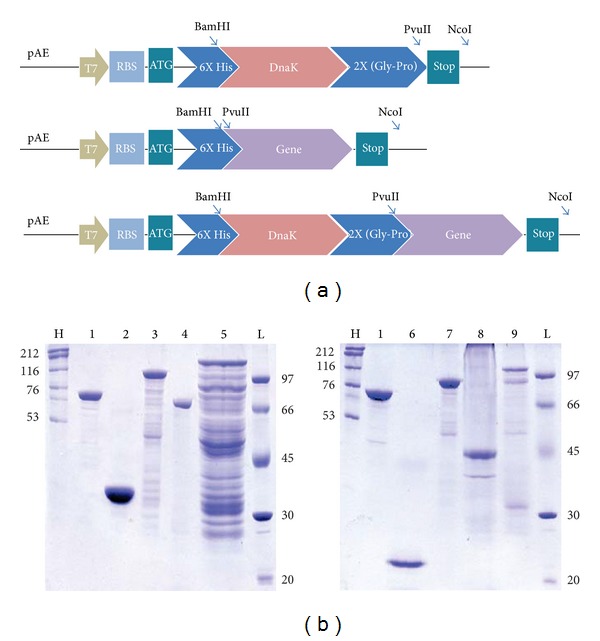
(a) Schematic representation of the expression cassettes. The genetically fused genes were obtained from individually cloned genes in pAE vector, and, then, surface protein genes were digested at PvuII/NcoI restriction sites and ligated in the same sites into pAE-DnaK construct. Depicted are T7 phage RNA polymerase promoter, ribosome-binding site (RBS), ATG start códon, 6X Histidine tag, restriction cloning sites, and the 2X (Gly-Pro) flexible hinge. (b) Analysis of purified recombinant proteins by SDS-PAGE. Purified recombinant protein eluted from Ni^+2^-charged Sepharose column with 1 M imidazole are visualized by Coomassie blue staining. Lane H (HMW) and L (LMW): high and low molecular mass protein markers; In kDa: lane 1: DnaK (68.9); lane 2: rLIC10494 (25.1); lane 3: DnaK-rLIC10494 (92.5); lane 4: rLIC12730 (75.7); lane 5: DnaK-rLIC12730 (143.1); lane 6: Lsa21 (19.8); lane 7: DnaK-Lsa21 (87.2); lane 8: Lp95 C-terminal (43.6); lane 9: DnaK-Lp95 C-terminal (110.9).

The genes, DnaK, LIC10368, LIC10494, LIC12730, and LIC12690, were amplified from* L. interrogans* serovar Copenhageni genomic DNA with complementary primers, designed with the restriction sequences at forward and reverse directions. Table 1 summarizes gene locus, recombinant protein given name, NCBI reference number, genome annotated protein function, sequence of the primers with the restriction cloning sites, and predicted molecular mass of the individual recombinant protein. The genes were first individually cloned into pAE plasmid. The construction generated with pAE-DnaK allows the directional cloning of other guest DNA inserts in fusion at the carboxy-terminus of DnaK gene. The fused proteins have 6X His tag at N-terminus of DnaK and a flexible 2X (Gly-Pro) hinge region incorporated between the C-terminus of DnaK and the N-terminus of the selected gene. The hinge purposes to separate the two components of the protein fusion, thus allowing each to undergo folding without steric hindrance from the other [22]. The scheme of the expression cassettes of the constructs is depicted in Figure 1(a).

### 3.2. Expression and Purification of Recombinant Proteins

**Figure 2:**
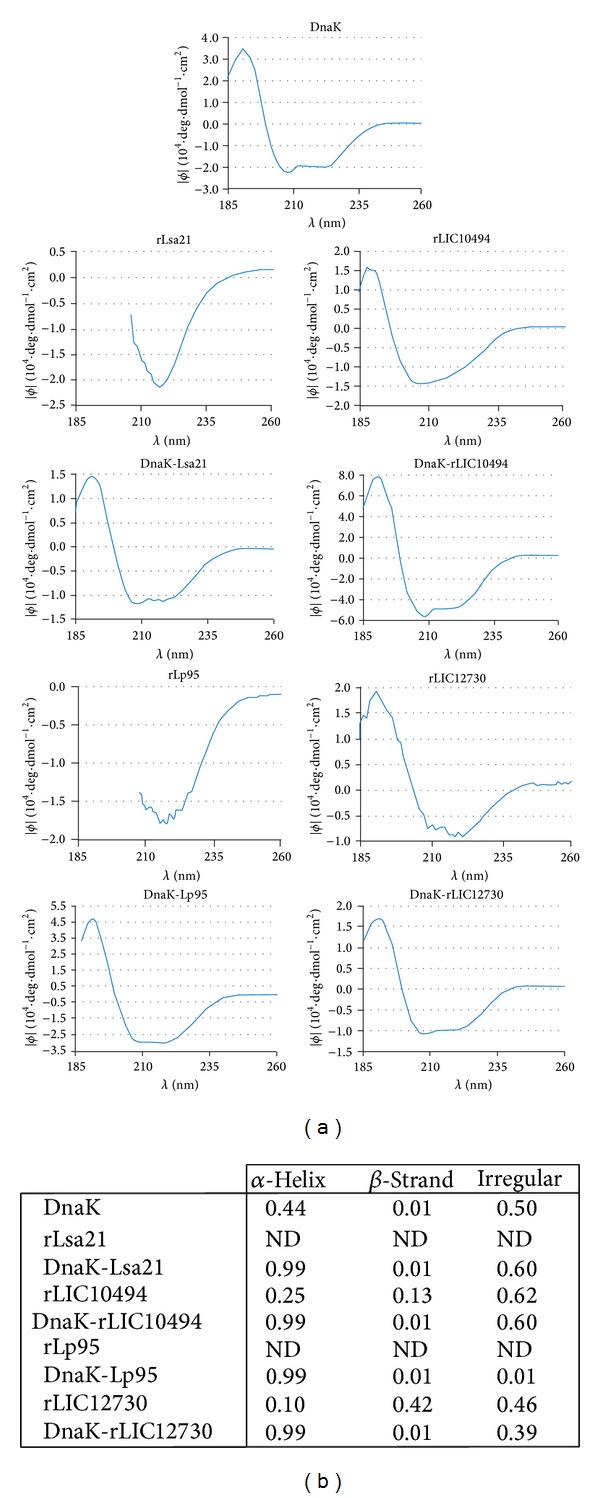
CD spectra of purified recombinant proteins depicted after refolding. (a) CD spectra (FAR-UV CD) of 10 *μ*M of each recombinant protein in 10 mM Na-phosphate buffer (pH 7.4), except Lsa21 and Lp95 C-terminus region that were in 100 mM Tris (pH 12.0) 500 mM NaCl, performed at 20°C. Far-UV CD spectra are represented as an average of five scans recorded from 185 to 260 nm. Ellipticity (*ϕ*) is expressed in function of wavelength. (b) Percentage of secondary structure of the recombinant proteins according to the analysis of CD spectra data by the CAPITO software. ND: not determined.

Genetically combined proteins were successfully obtained and high expression level of recombinant proteins was achieved using the pAE vector and* E. coli* BL21 (SI) (Figure 1(b)). DnaK and rLIC12730 proteins were expressed in a soluble form and purified from the supernatant. The other proteins were expressed in insoluble form, as inclusion bodies, and were purified after solubilization in 8 M urea and on-column chromatography refolding on Ni^2+^-charged beads. The expected recombinant protein bands either individually or fused with DnaK are visualized by Coomassie blue stained SDS-12% PAGE and shown in Figure 1(b): DnaK (69 kDa), rLIC10494 (25 kDa), DnaK-rLIC10494 (93 kDa), rLIC12730 (76 kDa), DnaK-rLIC12730 (143 kDa), Lsa21 (20 kDa), DnaK-Lsa21 (87 kDa), C-terminal portion of Lp95 (44 kDa), and DnaK-Lp95 C-terminal (111 kDa). Structural integrity of the purified proteins was assessed by circular dichroism (CD) spectroscopy. The method evaluates the secondary structure content of the protein and it is an important data to obtain after protein refolding. The CD spectrum of individually cloned and expressed protein and the spectrum of the corresponding fusion with DnaK are shown in Figure 2(a). Analysis of the spectrum data by CAPITO software is depicted in Figure 2(b), except for rLsa21 and Lp95 proteins that had their spectra badly resolved with very low absorption values. The data shows that DnaK, rLIC10494, and rLIC12730 secondary structure contents present a predominance of alfa-helix, the minima at 208 and 222 nm, and the maxima at 192 nm. The DnaK fusion proteins have had their secondary structure contents shifted to alfa-helix, similar to the DnaK spectrum alone. CD spectrum of DnaK presented higher absorption values than the ones of the proteins alone and has, probably, superimposed the CD spectra of the leptospiral proteins.

### 3.3. Antibody Response in Mice Immunized with the Recombinant Fused Antigens

**Figure 3:**
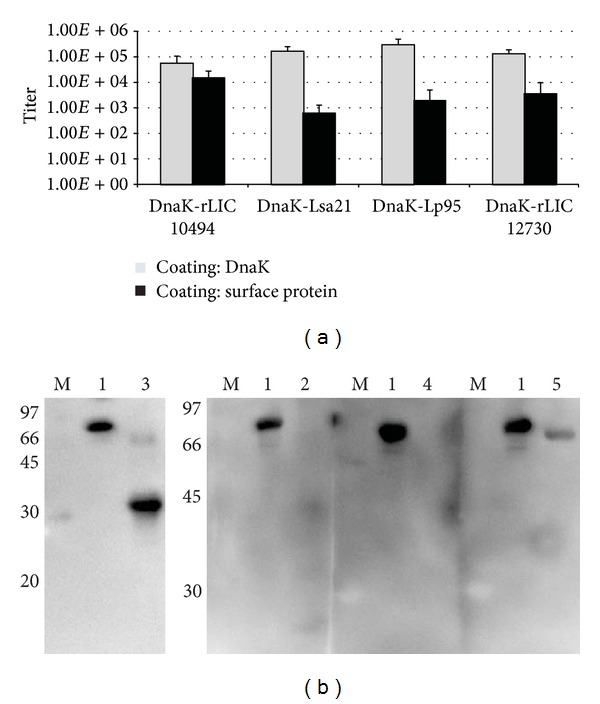
Analysis of IgG production induced in mice by recombinant proteins. The sera from mice immunized with fused-proteins were analyzed by ELISA (a) and Western blotting (b). In (a) wells were coated with DnaK or surface proteins, as depicted; in (b) blotted proteins are DnaK (lane 1), Lsa21 (lane 2), rLIC10494 (lane 3), Lp95 C-terminus region (lane 4), and rLIC12730 (lane 5). M: molecular mass protein marker. In both methods, proteins were probed with the respective antifusion protein serum (1 : 20,000 dilution) and the reactions were developed with HRP-conjugated anti-goat IgG (1 : 10,000) (ELISA) and HRP-conjugated anti-mouse IgG (1 : 5,000) (Western).

Total IgG antibody elicited in mice by fused-proteins was analyzed by ELISA (Figure 3(a)) and Western blotting (Figure 3(b)). The DnaK and the leptospiral proteins were used to individually coat ELISA microplates. The immune sera from mice injected with the fusion proteins, DnaK-Lsa21, DnaK-rLIC10494, DnaK-Lp95 C-terminal region, and DnaK-rLIC12730 were employed to probe the respective coated protein. The ELISA data show that mice responded with IgG antibodies against both proteins (Figure 3(a)). These results were strengthened by Western blotting data (Figure 3(b)), which show that both blotted proteins are individually recognized by their corresponding mice antisera injected with the fusion proteins, except for the proteins Lsa21 (DnaK-Lsa21) and Lp95 C-terminal region (DnaK-Lp95), where the place of the expected bands are indicated by symbols (*).

### 3.4. Evaluation of Total IgG and Subclasses in Sera of Mice Immunized with Recombinant Protein and DnaK Fused Proteins

**Figure 4:**
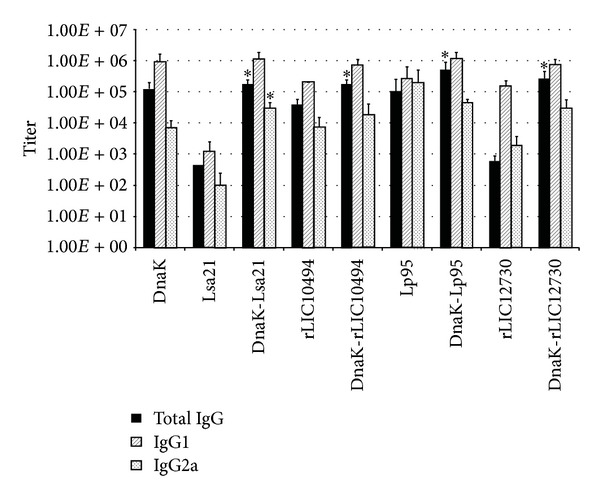
Analysis of IgG isotype profile in serum of mice immunized with the recombinant proteins. Sera from BALB/c mice immunized with the recombinant proteins alone or in fusion with DnaK were analyzed by ELISA. IgG, IgG1, and IgG2a titers were evaluated in each case. Sera from PBS injected mice were employed as negative control. Statistical analyses were performed by two-tailed *t*-test, comparing the titer obtained with leptospiral surface protein alone with the corresponding DnaK fusion protein.

In order to studythe type of immune response triggered in mice by the individual DnaK, Lsa21, rLIC10494, Lp95 C-terminus region, rLIC12730, and the corresponding fusion proteins with DnaK, we performed the ELISA using subclass-specific antibodies IgG1 and IgG2a. The results show that DnaK alone elicits a strong statistically significant IgG immune response, with a predominance of IgG1 (Figure 4), when compared to PBS-immunized control (data not shown). Lsa21 alone was not able to induce an efficient immune response in mice, but the DnaK-Lsa21 protein had an improved, statistically significant production of both IgG total and IgG2a subclass, when compared to Lsa21 alone (Figure 4). Analysis of proteins rLIC10494, Lp95 C-terminus, and rLIC12730 alone and with its DnaK fusion showed an increase, statistically significant titer values of total IgG. The values obtained with IgG subclasses were not statistically significant.

### 3.5. Evaluation of Lymphocyte Proliferation in Spleen Cells of Mice Immunized with Recombinant Proteins

**Figure 5:**
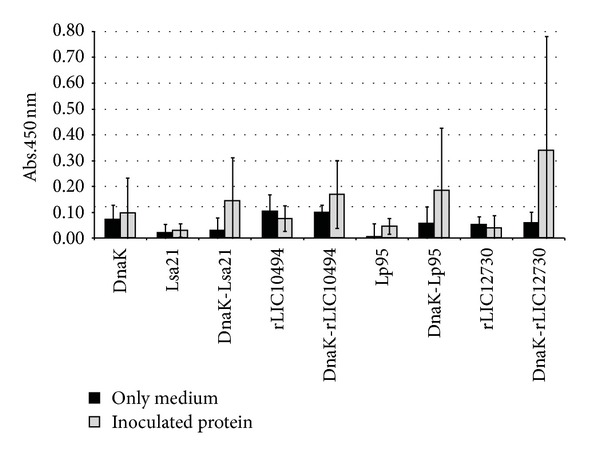
Analysis of lymphocyte proliferation in cultured splenocytes from mice immunized with recombinant proteins. Splenocytes from immunized mice were isolated and cultured for 48 h. The cells were stimulated with the recombinant protein alone or in fusion with DnaK. Cells were further incubated with BrdU and DNA synthesis was quantified by BrdU immunodetection kit (M&M). ConA and culture medium were employed as positive and negative stimulation controls, respectively (not shown). The data are from two independent experiments.

To evaluate the effect of proteins in fusion with DnaK on the cellular immune response of the recombinant protein alone, mice were immunized with the recombinant proteins, individually or in fusion with DnaK. Spleens from PBS-injected mice were employed as controls. Lymphocyte proliferation showed an increased, boosted effect when animals were immunized with fusion proteins compared with leptospiral proteins alone (Figure 5). In all cases, but remarkably with DnaK-rLIC12730, an improvement was observed when animals were primed and stimulated with proteins in fusion with DnaK (Figure 5), having a stimulation index of 8.72. DnaK-Lsa21, DnaK-rLIC10494, and DnaK-Lp95 C-terminus showed stimulation index of 4.89, 2.25, and 4.09, respectively. High proliferation level was obtained when cells were treated with ConA, employed as positive control of the experiment (not shown). Addition of proteins, either alone or in fusion with DnaK to lymphocytes from animals that have not been primed with either proteins or the corresponding DnaK fusions, produced nonsignificant levels of proliferation (data not shown).

### 3.6. Cytokine Production Evaluation in Splenocytes of Mice Immunized with Recombinant Proteins

**Figure 6:**
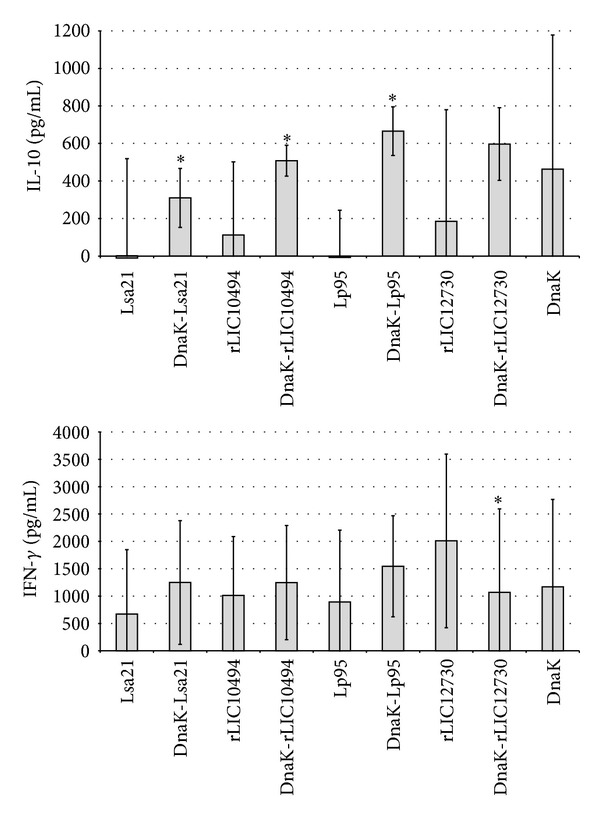
Analysis of cytokine profiles from spleen cells supernatant. Spleen cells from immunized mice were isolated and cultured in 24-well tissue culture plates. After 48 h, cell-free culture supernatants were collected, and the level of cytokines, IFN-*γ* and IL-10, was measured by ELISA. For statistical analysis, concentration values for the recombinant fusion proteins-immunized group stimulated with the same protein were compared with those immunized and stimulated with the corresponding protein alone by the two-tailed *t*-test.

In order to assess secreted cytokines, supernatants of cultured spleen cells from recombinant proteins immunized mice were analyzed for the presence of IL-10, IL-4, IFN-*γ*, and TNF-*α*, selected to discriminate Th1 (IFN-*γ* and TNF-*α*) and Th2 (IL-10 and IL-4) immune responses [31, 32]. Recombinant proteins Lsa21 and Lp95 C-terminal were not capable to promote secretion of IL-10 and the amount detected were similar to the controls (Figure 6). Proteins rLIC10494 and rLIC12730, alone, induced small amounts of IL-10, statistically significant compared to the controls (cell culture medium). A booster effect was elicited by the presence of DnaK in the protein fusions, being statistically significant with the proteins Lsa21, rLIC10494, and C-terminus of Lp95. The presence of DnaK does not promote an enhancement on IFN-gamma level, and in the case of rLIC12730, a decrease in the amount of IFN-gamma detected. Measurements of the same parameters with spleen cells from control animals immunized with medium either stimulated or not with the recombinant proteins produced negligible results (not shown). Cytokines IL-4 and TNF-alfa evaluation resulted in very low values (not shown).

## 4. Discussion

Heat shock proteins (HSPs) are a large family of proteins with diverse molecular mass and intracellular localizations. These proteins assume important functions in maintaining cell homeostasis, and they have, therefore, been conserved during evolution. Under physiological conditions, some of these proteins operate as intracellular molecular chaperones. Chaperones take part in the assembly, stabilization, folding, and translocation of oligomeric proteins. The expression of many HSPs is upregulated under stress conditions, nutritional deficiencies, ultraviolet irradiation, chemicals, viral infection, and ischemia-reperfusion injury [33, 34]. Chaperones, like bacterial DnaK and GroEl, have been reported to decrease recombinant protein aggregates and to assist their folding in* E. coli* host expression systems [35, 36]. In our studies, only Dnak and rLIC12730 were expressed in their soluble form. However, after refolding, all fusion proteins have their secondary structure contents well defined, consistent with the chaperone activity due to the presence of DnaK.

HSPs have immunological functions: they are highly immunogenic in BALB/c mice and recombinant complexes of antigens with DnaK have been reported to be CD8^+^ T-cell-stimulating immunogens [14, 37]. Studies performed with* Mycobacterium tuberculosis* heat shock fusion protein have shown to increase delivery and cross-processing by B lymphocytes, which could represent a new contribution to the generation of CD8^+^ T-cell responses [33].

It has been demonstrated that pathogenic leptospires can elicit Th1 response together with anti-lipopolysaccharide antibodies in animal model [38]. OmpL1 and LipL41 combined B- and T-cell epitopes can promote a Th1 immune response in BALB/c mice [39]. We have previously shown that OmpL1 of* L. interrogans* stimulated both Th1 and Th2 immune response in BALB/c mice [40]. More recently, we have reported that two leptospiral adhesins, Lsa44 and Lsa45, were capable of inducing a combination of humoral and cellular immune response in mice, characterized by high concentration of antibodies, induction of cellular lymphoproliferation and increased level of cytokines [41].

We have previously characterized four leptospiral proteins that we have expressed in* E. coli* system. Two of them were described as novel adhesins, Lsa21 and Lp95 C-terminus region [18, 19], and the other two, rLIC10494 and rLIC12730, were shown to have a modest effect in immunoprotection studies [20]. The leptospiral genes, DnaK, LIC10368, LIC10494, LIC12730, and LIC12690, were genetically cloned in fusion with DnaK. The DnaK-leptospiral fusion proteins, as well as, the proteins alone, were employed to immunize mice. The presence of DnaK in fusion with the leptospiral proteins Lsa21, rLIC10494, Lp95 C-terminus, and rLIC12730 promoted a boosted effect on their immunogenic activities either at humoral or cellular level, as denoted by an enhancement of total IgG production, induction of cellular lymphocyte proliferation and IL-10 increased levels in BALB/c mice. This improved effect is particularly evident with the recombinant Lsa21, a protein that has been previously shown to be present only in low passage, virulent strain of* Leptospira*, with a possible role in pathogenesis [18]. Similar to the data we have previously obtained with the adhesin Lsa44 of* Leptospira* [41], these fusion proteins did not induce either TNF-alfa or IL-4. IL-10 was described to have a special role in restraining and preventing an excessive immune response and collateral damage [42]. In the case of leptospirosis, it was observed that the induction of IL-10 is higher in mice than in hamsters animal model, consistent with the chronically, asymptomatic nature of this model [43]. Future studies will have to be conducted in hamsters in order to evaluate the ability of the recombinant antigens to elicit protective immune response in a susceptible species.

Taken together, our data show that leptospiral proteins in fusion with DnaK promoted an enhanced cellular and humoral immune response in mice. HSPs, such as prokaryotic DnaK, incorporated to antigens to boost their immunological properties, have potential advantages for use in vaccine development against leptospirosis.

## Figures and Tables

**Table 1:** Gene locus, protein given name, NCBI reference sequence number, features, sequence of the primers employed for DNA amplification, and molecular mass of expressed recombinant proteins.

Gene locus^1^	Recombinant protein given name	NCBI reference sequence number^2^	Description/function	Sequence of primers for PCR amplification and restriction cloning sites	Recombinant protein molecular mass
LIC10524	DnaK	YP_000508	Molecular chaperone DnaK	F: 5′GGATCCGCTATGGAAGGTGGAGACCCGGTC3′ (BamHI) R: 5′CCATGG ** ** GATATC TCACAGCTG CGGACCCGGACCCTTTTTCTCATCGTCTAC3′ (NcoI, EcoRV and PvuII)	68.9 kDa

LIC10368	Lsa21^a^	YP_000355	Putative lipoprotein	F: 5′GGATCC ** ** CAGCTG GCTATGG CCTGTCCGGATGAAAAAAAAG3′ (BamHI and PvuII) R: 5′CCATGG ** ** GATATCTCAAAATACATTCACACGAATATCTC3′ (NcoI and EcoRV)	19.8 kDa

LIC10494	rLIC10494^b^	YP_000478	Putative lipoprotein	F: 5′GGATCC ** ** CAGCTG GCTATGG TAATCTTTGGCAACAAAAC3′ (BamHI and PvuII) R: 5′CCATGG ** ** GATATC TCA TTTGTTTTCAGATTTTTTC3′ (NcoI and EcoRV)	25.1 kDa

LIC12690	Lp95 c-terminal^c^	YP_002611	Putative lipoprotein with a domain of unknown function (DUF1554)	F: 5′GGATCC ** ** CAGCTG GCTATGG AAAATGGAGAAGACGTTAC3′ (BamHI and PvuII) R: 5′CCATGG ** ** GATATC TCA TTGTTCCACACAAAGAATG3′ (NcoI and EcoRV)	43.6 kDa

LIC12730	rLIC12730^d^	YP_002650	Hypothetical protein with TPR motif	F: 5′GGATCC ** ** CAGCTG GCTATGG GACTTCCCAATTTCTCTATTC3′ (BamHI and PvuII) R: 5′CCATGG ** ** GATATC TCA TTTCGTAAAAAATCCAGCTTC3′ (NcoI and EcoRV)	75.7 kDa

^1^ http://aeg.lbi.ic.unicamp.br/world/lic/ [25].

^2^ http://www.ncbi.nlm.nih.gov/protein/. Protein BLAST-http://www.ncbi.nlm.nih.gov/blast/Blast.cgi [26, 27].

^a^Atzingen et al., 2008 [18], Atzingen et al., 2012 [28].

^b^Atzingen et al., 2010 [20], Vieira et al., 2010 [29].

^c^Atzingen et al., 2009 [19].

^d^Atzingen et al., 2010 [20], Vieira et al., 2010 [29], Felix et al., 2011 [30].
